# A New Tumor Delineation Method for Brain Metastases Radiotherapy by Jointly Referring to Contrast-Enhanced T1-Weighted and Fluid-Attenuated Inversion Recovery MRI

**DOI:** 10.7759/cureus.9106

**Published:** 2020-07-10

**Authors:** Maki Soyama, Rieko Azumi

**Affiliations:** 1 Radiology, Nishi-Niigata Chuo National Hospital, Niigata, JPN

**Keywords:** mri, contrast-enhanced t1-weighted, contrast-enhanced fluid-attenuated inversion recovery, ce-3d-flair, whole brain radiotherapy, wbrt, simultaneous integrated boost, sib, brain metastases, volumetric modulated arc therapy

## Abstract

A new tumor delineation technique for brain metastases has been proposed by jointly referring to thin-slice contrast-enhanced T1-weighted and thin-slice contrast-enhanced fluid-attenuated inversion recovery magnetic resonance (MR) images. A single-isocenter six-arc noncoplanar volumetric modulated arc radiotherapy (VMAT) plan for 16 brain metastases was created by the Monaco treatment planning system (Elekta AB, Stockholm, Sweden) with a photon energy of 6 MV. Each gross target volume (GTV) was very carefully delineated on all three orthogonal planes of the above two different MR images. A dose of 37.5 Gy was prescribed to 96% of the whole brain in 15 fractions with a simultaneous integrated boost (SIB) dose of 57 Gy to 95% of each of the eight GTVs each having a volume larger than 0.05 cm^3^ and another SIB dose of 52.5 Gy to 90% of each of the remaining eight smaller GTVs. For accurate tumor localization, an in-house thermoplastic mask was developed by modifying a commercial thermoplastic shell, in such a way that a portion of the thermoplastic shell was pushed into a patient mouth so that the patient can bite it with the lips and the teeth. The outer cylinder of a syringe was additionally pushed into the resulting mouthpiece portion, thereby providing an air duct for easier mouth breathing. Immediately before the VMAT delivery, bone matching was performed between planning CT and on-board cone-beam CT images; thereafter, a six-degrees-of-freedom couch was activated to correct the translational and rotational set-up errors. The treatment time per fraction was approximately 30 minutes including the couch rotations.

## Introduction

Improved systemic cancer treatment has resulted in prolonged survival of patients with brain metastases, thereby demanding long-term local control after the treatment course is completed. The brain metastases are usually delineated by referring to contrast-enhanced 3D T1-weighted (CE-3D-T1W) MRI, which is subsequently co-registered to the planning CT [1]. Recently, the importance of contrast-enhanced 3D fluid-attenuated inversion recovery (CE-3D-FLAIR) MRI was demonstrated in various intracranial pathological disorders by comparing with CE-3D-T1W [2,3]. However, to the author’s best knowledge, CE-3D-FLAIR has not been used for delineating brain metastases in radiotherapy planning. We have proposed an arguably more accurate tumor delineation technique for brain metastases by jointly referring to CE-3D-T1W and CE-3D-FLAIR images. In our institution, radiotherapy regimens for brain metastases from non-small cell lung cancer depend on the number of metastases: stereotactic radiotherapy (SRT) for oligometastases and whole brain radiotherapy (WBRT) with simultaneous integrated boost (SIB) for 10 or more metastases. We report a case of the latter, where the number of metastases is 16.

## Case presentation

A 69-year-old female patient was diagnosed with epidermal growth factor receptor (EGFR) mutation-positive non-small cell right lung adenocarcinoma (cT2aN3M0) in October 2017. Chemoradiotherapy was applied, and the following consolidation treatment was interrupted after two doses due to hepatic disorders. In July 2018, the patient was diagnosed with multiple brain metastases and multiple liver metastases, and she was treated with EGFR tyrosine kinase inhibitor (TKI) leading to significantly reduced tumor volumes of both metastases. The TKI therapy was terminated at the end of September due to lung toxicity. In January 2019, the brain tumor regrowth was observed and radiotherapy was ordered. In February 2019, WBRT with SIB to 16 brain metastases was performed using volumetric modulated arc radiotherapy (VMAT). Written informed consent was obtained under the patient’s performance status of 1.

A single-isocenter six-arc noncoplanar VMAT plan with a photon energy of 6 MV was created for the 16 brain metastases by the Monaco treatment planning system (Elekta AB, Stockholm, Sweden) [4,5]. Each gross target volume (GTV) was very carefully delineated on all three orthogonal planes using thin-slice CE-3D-T1W and thin-slice CE-3D-FLAIR MR images being further fused to the patient CT images. In this paper, the thin slice refers to a slice having a thickness of 1.4 mm. For the contrast enhancement, Gadovist (Bayer AG, Leverkusen, Germany) at a dose of 0.1 mL/kg was administered by intravenous bolus injection. A dose of 37.5 Gy was prescribed to 96% of the whole brain in 15 fractions with an SIB dose of 57 Gy to 95% of each of the eight GTVs each having a volume larger than 0.05 cm^3^ and another SIB dose of 52.5 Gy to 90% of each of the remaining eight smaller GTVs.

For accurate tumor localization, an in-house thermoplastic mask was made by modifying a commercial thermoplastic shell, in such a way that a portion of the thermoplastic shell was pushed into a patient mouth so that the patient can bite it with the lips and the teeth [6]. In addition, the outer cylinder of a syringe was pushed into the resulting mouthpiece portion, thereby providing an air duct for easier mouth breathing. Immediately before the VMAT delivery, bone matching was performed between the planning CT and the on-board cone-beam CT. Subsequently, a six-degrees-of-freedom couch was activated to correct the translational and rotational set-up errors. The planned dose was delivered by a Synergy linac (Elekta AB, Stockholm, Sweden).

**Figure 1:**
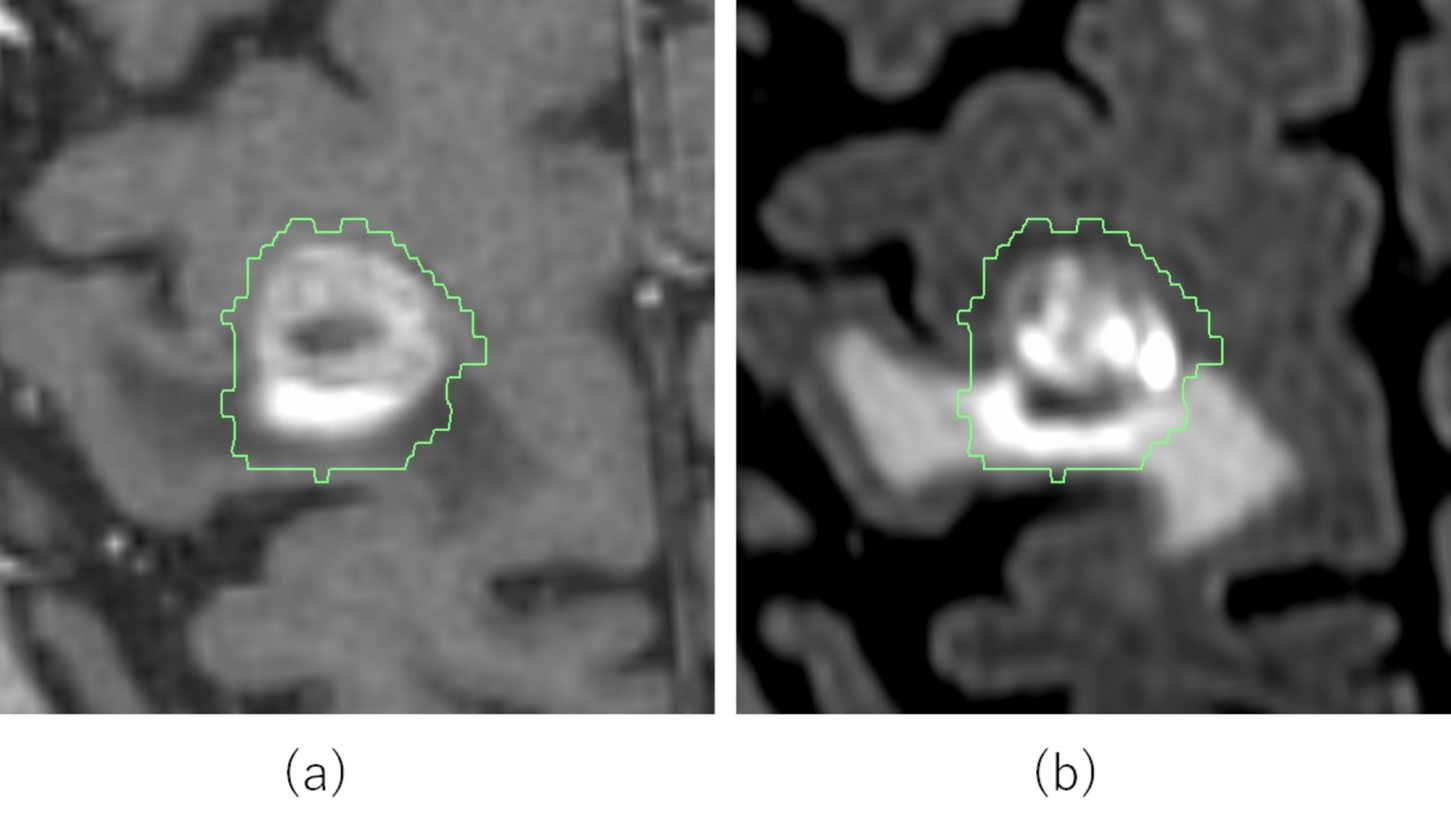
Delineation of gross tumor volume by jointly referring to (a) thin-slice contrast-enhanced 3D T1-weighted (CE-3D-T1W) and (b) thin-slice contrast-enhanced 3D fluid-attenuated inversion recovery (CE-3D-FLAIR) magnetic resonance images.

Figure 1 shows the delineated GTV by jointly referring to two different images of (a) thin-slice CE-3D-T1W and (b) thin-slice CE-3D-FLAIR, where the CE-3D-FLAIR provides an additional enhanced area that expands outward from the enhanced area in the CE-3D-T1W.

**Figure 2:**
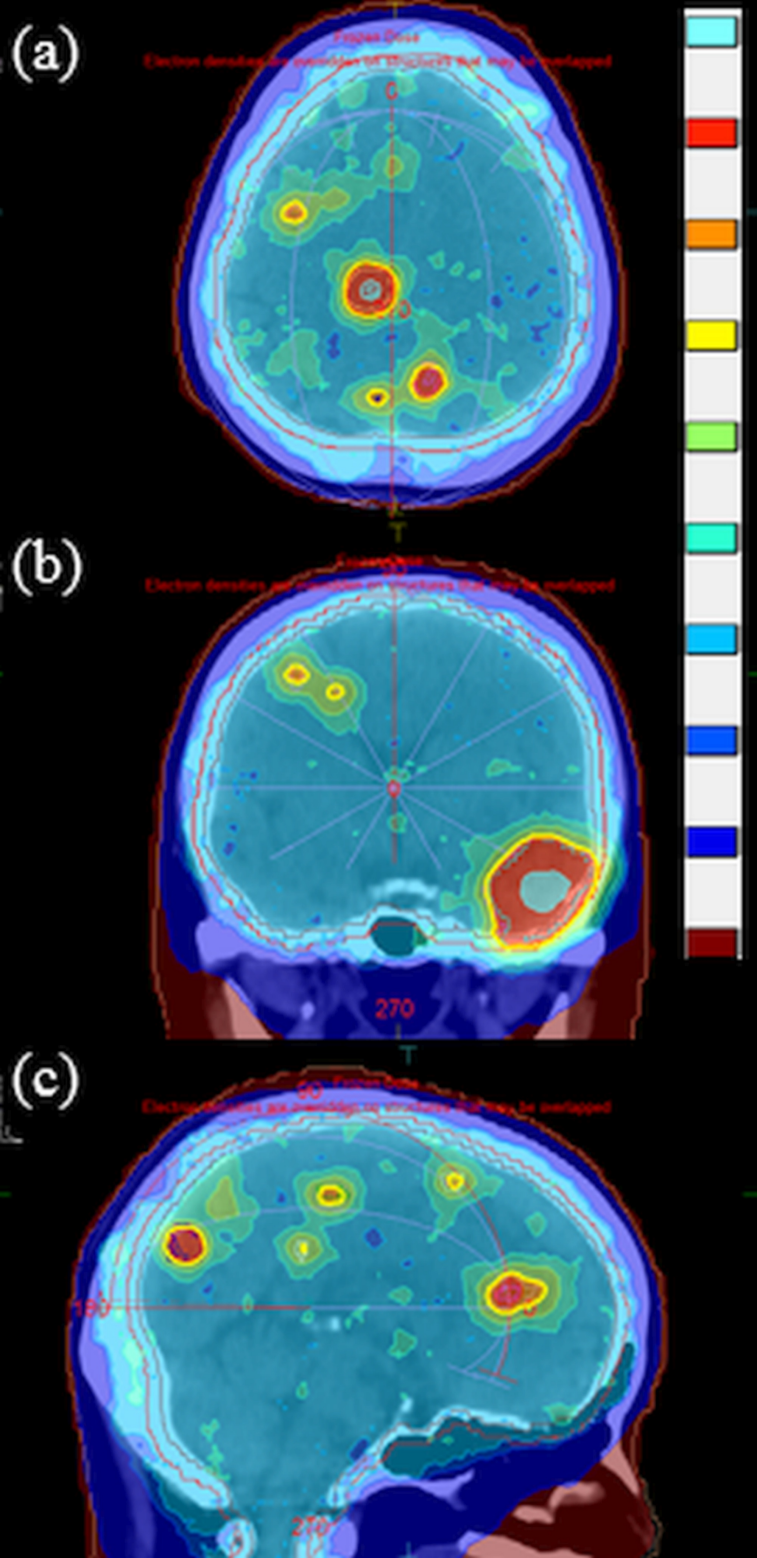
Dose distributions on (a) an axial plane, (b) a coronal plane, and (c) a sagittal plane for the 16 brain metastases. The color bar (from top to bottom) shows dose thresholds of 80, 57, 54, 52.5, 46, 41.25, 37.5, 35.63, 20, and 6 Gy.

Figure 2 indicates the optimized dose distributions for the 16 brain metastases.

**Figure 3:**
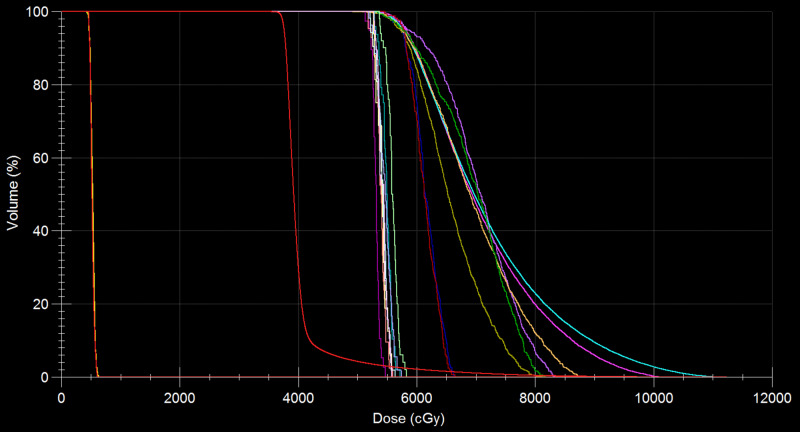
Calculated dose-volume histograms (DVHs). The eight curves on the right-hand side show the DVHs of the major eight gross target volumes (GTVs) each having a volume larger than 0.05 cc. More closely populated eight curves to the left-hand side of the aforementioned eight curves indicate the DVHs of the remaining smaller eight GTVs. Further to the left, the three steep curves correspond to the DVHs of the whole brain and then each of the eye lenses at the left end.

Figure 3 demonstrates the dose-volume histograms (DVHs) resulting from the optimized dose distributions shown in Figure 1.

**Figure 4:**
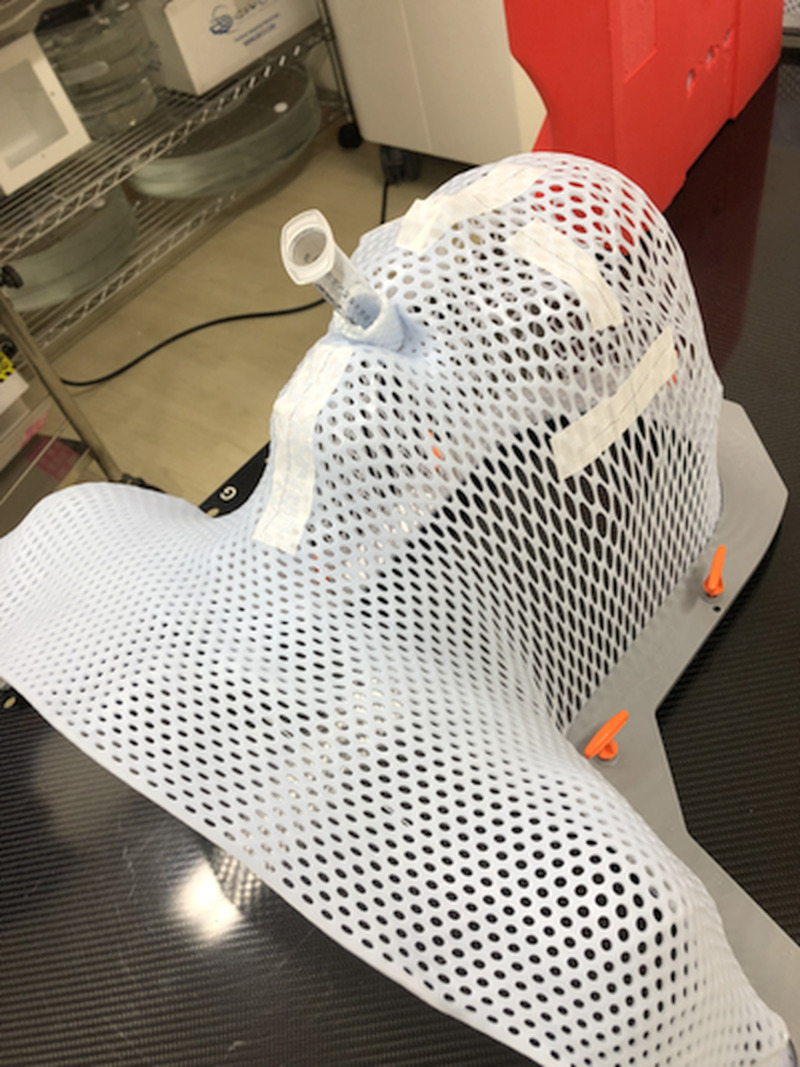
A thermoplastic mask having an in-house integrated mouthpiece. The mouthpiece portion was made by pushing the pre-heated thermoplastic mask into the patient mouth. Immediately after creating the mouthpiece portion, the outer cylinder of a syringe was further pushed into the mouthpiece portion thereby providing an air duct for easier mouth breathing.

Figure 4 shows a thermoplastic mask with an in-house integrated mouthpiece. The mouthpiece portion was made by pushing the pre-heated thermoplastic mask into the patient mouth. The treatment time per fraction was approximately 30 minutes including the couch rotations.

After initial dose delivery, the patient showed temporary disorders of consciousness similar to symptomatic epilepsy and thus required hospitalization. The treatment continued on the next day onwards due to reduced symptoms. On the last day of the treatment, most of the neurological symptoms disappeared thereby allowing the patient to walk home.　 

## Discussion

The challenges of the WBRT with SIB are (1) the threshold dose causing brain necrosis may be relatively low compared to SRT alone, and therefore, the prescribed total dose to the tumor may need to be reduced compared to that for the SRT alone, and (2) a change in a tumor-associated edema volume may result in tumor displacement, thereby requiring recontouring and adaptive planning during the course of treatment. However, it is still arguable whether the GTV contours should be shrunk when the tumor volume is visually reduced during the treatment, considering that microtumors may surround the tumor body.

In this study, each GTV was very carefully delineated on all three orthogonal planes by referring to both of thin-slice CE-3D-T1W and thin-slice CE-3D-FLAIR images. This is because the CE-3D-T1W alone may not accurately identify tumor-infiltrated normal brain regions. Zhou et al. reported that the size and the extent of lesions on the CE-FLAIR images were significantly larger than those on the CE-T1W images and that the results of CE-FLAIR images were highly correlated with the surgical findings [2]. Lee et al. explained this observation with the following hypothesis: CE-3D-FLAIR provides image enhancement at regions of low gadolinium concentrations, whereas CE-3D-T1W provides image enhancement inside the tumor where gadolinium concentrations are high [3]. It is the present authors’ opinion that a region of low gadolinium concentrations may be associated with tumor-infiltrated normal brain where the blood-brain barrier is less disrupted. CE-3D-FLAIR may therefore be able to directly locate the tumor-infiltrated normal brain region. Although Siam et al. observed clear boundaries between brain metastases and surrounding normal brain tissues, Baumert et al. reported that infiltrative growth beyond the border of the brain metastasis was pathologically demonstrated in 63% of the cases evaluated [7,8]. It is also well known that the above tumor infiltration is a major cause of local recurrence after surgical tumor resection. 

In our institution, each GTV for the brain metastases includes the enhanced areas in the CE-3D-FLAIR images. As a result, GTV contours for the present case are partly expanded outwards approximately by 1 mm compared to more traditional contours based on CE-3D-T1W alone. Noël et al. added an isotropic margin of 1 mm to an enhanced area of CE-T1W in their linac radiosurgery with a Leksell frame (Elekta AB, Stockholm, Sweden), reporting an improved local control [9]. Nataf et al., on the other hand, showed that a margin of 2 mm did not increase the local control while increasing severe parenchymal complications [10]. These previous findings may also support our anisotropic 1 mm margin strategy based on CE-3D-FLAIR images.

When we observe an enhanced area in CE-3D-FLAIR image that expands outward from the corresponding CE-3D-T1W image, we often experience a peritumoral edema surrounding the enhanced area of the CE-3D-FLAIR image as shown in Figure 1b, and the enhanced area in short-term repeated CE-3D-FLAIR is highly reproducible. Conversely, no such enhanced areas are observed in CE-3D-FLAIR where the edema is insignificant. It is therefore highly speculated that the peritumoral edema may associate with tumor infiltration to the normal brain tissues. Thus far, no increase in radiation toxicity has been observed after employing this delineation procedure.

## Conclusions

We have proposed a new tumor delineation technique for brain metastases radiotherapy planning by jointly referring to thin slice CE-3D-T1W and thin slice CE-3D-FLAIR images, thereby arguably including tumor-infiltrated normal brain in the GTV.
